# The imperative to embed ethics in health research priority-setting

**DOI:** 10.2471/BLT.25.295304

**Published:** 2026-06-01

**Authors:** Joseph Millum, Bernardo Aguilera, Soumyadeep Bhaumik, Enrico Galvagni, Katherine Littler, Nchangwi Syntia Munung, Leah Pierson, Bridget Pratt

**Affiliations:** a Department of Philosophy, University of St Andrews, College Gate, North Street, St Andrews, Fife, KY16 9AJ, Scotland.; b Faculty of Medicine, Universidad San Sebastián, Santiago, Chile.; c Meta-research and Evidence Synthesis Unit, George Institute for Global Health, University of New South Wales, Sydney, Australia.; d Department of Philosophy, University of Edinburgh, Edinburgh, Scotland.; e Department of Research for Health, World Health Organization, Geneva, Switzerland.; f Faculty of Health Sciences, University of Cape Town, Cape Town, South Africa.; g Department of Internal Medicine, Brigham and Women's Hospital, Boston, United States of America.; h Queensland Bioethics Centre, Australian Catholic University, Brisbane, Australia.

Funding for health research has historically been insufficient to address global needs. However, recent cuts to national research budgets and foreign aid have raised alarms about the sustainability of funding for science.^1^^,^^2^ The expected shortfalls are unlikely to be fully compensated by other traditional research funding donors or private sector actors.^3^ Reduced global research budgets will force public and private funders, government ministries and research institutions to make difficult decisions about what health research to undertake. Trade-offs are inevitable. 

Currently, many research funders do not report on how they decide funding priorities. Those funders, governmental bodies and international actors who make their processes public use widely varying methods.^4^ Despite recent changes, many prioritization processes are still driven by technical experts rather than inclusive of patients, communities and health workers. Imbalances in the distribution of global health research persist decades after a report estimated that only 10% of global health research funds were directed at conditions that accounted for 90% of global disease burden.^5^^,^^6^

Health research priority-setting necessarily involves ethics because decisions about what research to support or conduct are essentially decisions about how to allocate scarce and valuable societal resources. Just as the allocation of health-care resources influences the distribution of health and illness, so do decisions about the allocation of health research resources. Therefore, priority-setting in health research, as in health care, is about justice.^7^

Recent guidance from the World Health Organization (WHO) offers a framework to embed ethics into research priority-setting, strategic planning and agenda-setting.^8^ The most important message of the new guidance is that research funders, research institutions and researchers have an ethical obligation to set research priorities in a rigorous, evidence-based and ethically defensible way.

The guidance articulates four broad ethical principles to guide priority-setting. First, optimize social value. The ultimate aim of health research should be to improve well-being and reduce inequity as much as possible. Research options should be compared using criteria that reflect the likelihood that the research will be successful, the anticipated benefits that might result and the impact on equity that those benefits would have. Second, respect special obligations. Research priorities should be consistent with the obligations of those who will support or conduct the research, such as those of researchers to the communities in which they work, and governments to their residents and the international community. Third, assess risks. Any reasonably predictable harms from research to populations who are not potential beneficiaries of the research should be assessed, minimized and justified. These include harms imposed on non-human animals during the course of research and potential harms to human third parties, as from research that is likely to have serious adverse environmental effects and dual-use research. Fourth, follow fair procedures. The priority-setting process that applies the other three ethical principles should be accountable, transparent and inclusive. Achieving inclusivity requires considering who should be included and whether participants can raise their voice and be heard at each stage of priority-setting. Particular attention should be paid to including members of disadvantaged and marginalized groups.^8^

These principles are both substantive and procedural: they highlight that ethical considerations should be built into both the process through which priorities are set and the outcomes of that process. Using a method that reflects these principles should lead to research agendas that are more responsive to global health needs and inequities and to the scientific opportunities to address them.

The guidance details how to incorporate ethical principles into priority-setting exercises, for those using existing methods as well as those designing their own. An accompanying casebook illustrates how different decision-makers have grappled with incorporating ethics into health research priority-setting.^9^

Importantly, the guidance does not prescribe a single approach to priority-setting. While an obligation to set priorities explicitly and ethically exists, the optimal approach depends on the context, timeframe and available financial and human resources. Hence, the guidance emphasizes that the time and resources devoted to priority-setting should be proportional to what is at stake.

In light of shrinking research budgets, one natural response will be to make the argument for why health research is important and deserves more support. However, ensuring that existing resources are used wisely is just as important. That means more than simply avoiding waste. Those who have decision-making power over what research is conducted have a responsibility to make their decisions on the basis of explicit, systematic and ethically informed priority-setting. This guidance will help them do so.
